# Surface Analysis of Orthodontic Mini-Implants after Their Clinical Use

**DOI:** 10.3390/jfb15090244

**Published:** 2024-08-24

**Authors:** Tamara Rahela Ioana, Filip George Boeru, Iulian Antoniac, Ioana Mitruț, Ionela Elisabeta Staicu, Anne Marie Rauten, Willi Andrei Uriciuc, Horia Octavian Manolea

**Affiliations:** 1 Department of Orthodontics, Faculty of Dental Medicine, University of Medicine and Pharmacy of Craiova, 200349 Craiova, Romania; tamigmg@yahoo.com (T.R.I.); elisabeta.staicu@umfcv.ro (I.E.S.); anne.rauten@umfcv.ro (A.M.R.); 2 Private Practice “Embrace”, 200618, Craiova, Romania; filipboeru@gmail.com; 3 Faculty of Material Science and Engineering, National University of Science and Technology Politehnica Bucharest, 060042 Bucharest, Romania; 4 Department of Dental Technology, Faculty of Dental Medicine, University of Medicine and Pharmacy of Craiova, 200349 Craiova, Romania; 5 Department of Oral Rehabilitation, Faculty of Dental Medicine, “Iuliu-Hațieganu” University of Medicine and Pharmacy, 400012 Cluj-Napoca, Romania; willi.uriciuc@umfcluj.ro; 6 Department of Dental Materials, Faculty of Dental Medicine, University of Medicine and Pharmacy of Craiova, 200349 Craiova, Romania; horia.manolea@umfcv.ro

**Keywords:** temporary anchorage devices, biocompatibility, orthodontic mini-implants, bacterial plaque, surface morphology changes, corrosion analysis

## Abstract

Temporary anchorage devices (TADs) are orthodontic mini-implants with remarkable characteristics that, once inserted, present mechanical retention (primary stability) without the process of bone osseointegration. However, interaction with the biological environment may cause changes in the morphology of the external surface of dental TADs. In this study, we used 17 TADs made of aluminum–vanadium titanium alloy, produced by two companies, which were analyzed through optical microscopy after being removed from the patients during orthodontic treatment. We evaluated the changes that appeared on the TADs’ surfaces after their use in the biological environment, depending on the morphological area in which they were inserted. In our study, we found changes in the morphology of the implant surface, and especially deposits of biological material in all study groups. On all samples examined after clinical use, regardless of the period of use, corrosion surfaces in different locations were observed. Our obtained results support the idea that the biological environment is aggressive for mini-implant structures, always producing changes to their surface during their clinical use.

## 1. Introduction

Modern orthodontics enjoys the remarkable capabilities of temporary skeletal anchorage devices (TADs) that have become a vital tool in the orthodontist’s hand. Improving treatment efficiency, patient compliance, and the performance of complex dental movements that could not previously be achieved without apparent side effects are some of the major advantages of using orthodontic mini-implants [1].

Temporarily fixed in bone, a temporary anchorage device (TAD) aims to improve orthodontic anchorage either by supporting the teeth of the reactive unit or by completely eliminating the need for the reactive unit, which is then removed after use. Orthodontic mini-implants (TADs) are composed of Ti-6Al-4V. Titanium is a highly reactive metal, which when exposed to fluid media or air quickly forms a titanium dioxide (TiO_2_) layer at the interface between the biological medium and metal structure and prevents further damage to the materials. This layer produces passivation of the metal, determining the degree of biocompatibility and biological response to the implant. Metal corrosion and a biocompatibility decrease are caused by any break in the oxide layer [2].

Two concepts predominate in which orthodontic mini-implants are used: one is the fixation of a dental anchorage unit by connecting it to the implant (indirect anchorage), and the other is the direct anchorage of the orthodontic mini-implant with reactive forces (direct anchorage). There is the need to identify and evaluate which of the two concepts is more beneficial, direct anchoring or indirect anchoring, and this is decided by the doctor depending on the clinical situation [3].

Orthodontic mini-implants are used for a limited period of time during orthodontic treatment and are then removed using a minimally invasive technique [4].

During orthodontic treatment, one of the most challenging aspects is the control of anchorage loss. Since the 1970s, some authors have described the use of osseointegrated implants to control tooth movement during orthodontic treatment in animal models, but it was not until 1997 that Kanomi first reported the use of orthodontic mini-implants to avoid this problem, and these have been widely used in clinical practice to date [5].

Introduced by Kanomi, orthodontic mini-implants, due to their low invasiveness, have become an established method of improving orthodontic anchorage [6].

Dento-maxillary anomalies have an ever-increasing prevalence, especially among adolescents; they cause growth disturbances at the level of the dental arches and are considered a public health problem. Dentoalveolar prominence is caused by an anomaly that is difficult to treat, and one of the objectives of the orthodontic treatment is to obtain an absolute anchorage. The easy placement and removal of orthodontic mini-implants has increased patient compliance, with a reported survival rate of 80% to 90%. Because orthodontic mini-implants are not attached directly to the teeth, and because after their placement they make close contact with the bone, they do not allow unnecessary movements and are considered anchoring systems that act as an “absolute” device [7].

For several years now, attention has been focused on orthodontic mini-implants due to their remarkable characteristics, which led to the completion of numerous studies that were carried out in various clinical situations. Some of the therapeutic procedures that can be performed with orthodontic mini-implants are dental extrusion and intrusion, closure of post-extraction spaces, and correction and leveling of the occlusion plane. Orthodontic mini-implants can also be combined with dental anchorage in cases of palatal expansion, distalization, mesialization, and correction of a class III malocclusion. The effectiveness of mini-implants has been demonstrated both in vitro and in vivo, and skeletal anchorage, combined with the advanced digital technology now available, has opened new horizons in orthodontic treatment, allowing for increasingly predictable results [8].

Orthodontic mini-implants (also called microscrews, micro/mini-implants, orthodontic implants, or TADs) are devices specially created to be inserted into the bone and to ensure anchorage. The advantage of these devices is the ability to fix, once inserted, the mechanical retention (primary stability) without the process of bone osseointegration occurring, which makes it simpler and less invasive to remove the device at the end of the orthodontic treatment [9].

The dental movements in the transverse, vertical, and anterior–posterior plane are only an obstacle for the doctor; they are carried out with the help of TADs, obtaining a quality therapeutic result without side effects. TADs have been shown to be well accepted by orthodontists and patients and are safe and effective treatment options in anchorage control. A recent study in Switzerland found that distalization with mini-implants was the most popular treatment plan selected by participating orthodontists [10].

Currently, mini-orthodontic implants are available in a number of different sizes. Each doctor chooses the length and diameter of the TAD, depending on what he wants to achieve clinically, but an advantage of the larger mini-implants is their capacity to distribute the applied force over larger areas of bone with less bone stress [11].

One of the frequent questions put by the patient to the orthodontist is how long the orthodontic treatment lasts. Patients undergoing fixed orthodontic therapy want to complete the treatment as quickly as possible, which is why it is essential that the orthodontist corrects the malocclusion without other associated iatrogenic problems. The use of temporary anchorage devices (TADs) has revolutionized fixed orthodontic therapy, allowing the doctor to plan a wide range of movements, which in the past could not be carried out so easily [12].

The null hypothesis of the study was that the interaction with the biological environment causes changes in the morphology of the external surface of dental TADs; therefore, the primary goal of this study was to analyze the external surfaces of the TADs after their removal from the biological environment at the time of fulfilling their medical purpose and to compare them with the surface appearance of the unused TADs.

The secondary goal was to evaluate the TADs’ morphology surface changes depending on the morphological area in which they were inserted, in order to identify a characteristic pattern of each area. In addition, we also investigated the chemical composition of the selected TAD areas both on new unused TAD surfaces and on clean used TAD surfaces, as well as on TAD surfaces covered by biological deposits.

## 2. Materials and Methods

For this study, we used 17 TADs made of aluminum–vanadium titanium alloy from two systems produced by two companies: Dual Top Anchor System (Jeil Medical Corporation, Seoul, Republic of Korea) and OrthAnchor System (Osstem, Eschborn, Germany). The TADs had a diameter of 1.4 mm, 1.6 mm, or 2 mm and a length of 6, 8, or 12 mm.

The mini-implants were bought from the Romanian market, and there was no involvement of the manufacturers.

Depending on the implant type, 2 control groups were defined, each group containing one new unused mini-implant from the 2 different systems.

Control Group 1: One new, unused TAD, where Dual Top Anchor System (Jeil Medical Corporation, Republic of Korea) was used. This TAD was used as a comparison sample.

Control Group 2: One new, unused TAD, where OrthAnchor System (Osstem, Eschborn, Germany) was used. This TAD was used as a comparison sample.

Their surfaces were analyzed firstly through optical microscopy, using the Nikon SMZ745T stereomicroscope. The surfaces were also analyzed with the help of scanning electron microscopy (SEM), using an FEI QUANTA INSPECT F microscope (FEI Company, Eindhoven, Netherlands) with energy dispersive spectroscopy.

Depending on the anatomical area in which they were inserted, the other 15 TADs were divided into 3 study groups, organized depending on the most common clinical indications.

Group 1—infrazygomatic-inserted TADs, where Dual Top Anchor System (Jeil Medical Corporation, Republic of Korea) was used. These TADs were used mainly for retrusion of the frontal teeth group.

Group 2—interdental-inserted TADs, where Dual Top Anchor System (Jeil Medical Corporation, Republic of Korea) was used. These TADs were used for several clinical indications like dental extrusion and intrusion and closure of post-extraction spaces.

Group 3—palatal vault-inserted TADs, where OrthAnchor System (Osstem, Eschborn, Germany) was used. These TADs were used mainly for expansion of the palatal vault.

The patients in this study were randomly selected; they needed these devices as temporary anchorage during corrective orthodontic treatment for several types of tooth movements, such as retraction of anterior teeth, molar distalization, tooth intrusion, and mesialization.

Exclusion criteria: patients with a poor general condition, smokers, patients younger than 18, and patients without adequate oral hygiene.

The mini-implants had been used clinically for 12 to 18 months; average use was 15.5 months. The clinical procedure for the insertion and use of all mini-implants was similar.

1. Insertion and removal

The TADs were inserted and removed by the same operator (F.B.), who followed the recommended insertion protocol for self-drilling TADs. At the end of each patient’s orthodontic treatment, TADs were removed according to the protocol. The mini-implants were inserted in the anatomical area corresponding to the study group of which they were a part.

2. Storage

After the devices were removed from the patients, they were stored in a 10% buffered formalin solution in plastic containers. We used an identification label for each sample and the records that contained them: patient name, sex, age, type of TAD with nominal dimensions of it, the clinical indication for their use, the place of insertion, the date of insertion and removal (the time of clinical use), and whether or not they had mobility at the time of removal.

3. Optical microscopy analysis

The surface morphology changes of the mini-implants were analyzed with the optical microscope using different magnification (10×–75×). At lower magnifications, the overall image of the mini-implant surface was analyzed, while at higher magnifications, the analysis was performed on 4 areas:

  − Z1—the surface of the mini-implant head that remains free in the oral cavity and to which the metal ligatures or elastic orthodontic wires are attached, a surface that is later covered with acrylate most of the time for the patient’s comfort.
  − Z2—the cervical surface (the transmucosal profile that will be covered by soft gingival–mucosal connective tissue) and the cervical third of the body (the active part inserted intraosseously).
  − Z3—the surface of the middle third of the body.
  − Z4—the surface of the apical third of the body—the TAD tip.

4. Scanning electron microscopy (SEM) analysis

The surfaces were also analyzed with the help of SEM (scanning electron microscopy), and in the areas where modifications of the TAD surfaces were highlighted, the EDX (Energy-Dispersive X-ray) analysis revealed the chemical composition present.

## 3. Results

*Control groups*

**Figure 1:**
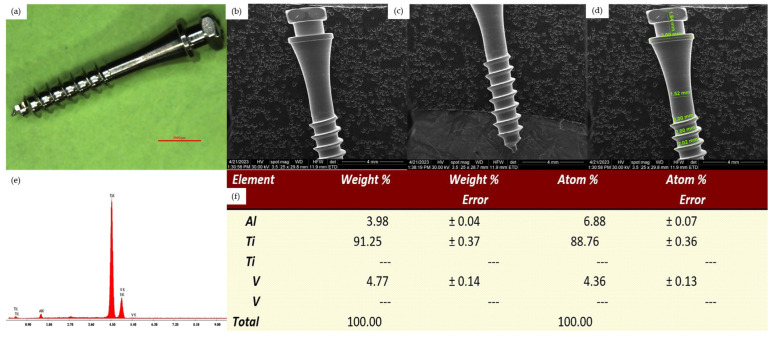
Surface analysis of an unused Dual Top Anchor System implant. (**a**) Overview aspect seen by optical microscopy, 10×. (**b**) SEM aspect of the cervical half, Z1-Z2 areas. (**c**) SEM aspect of the apical half, Z2-Z4 areas. (**d**) SEM aspect of the cervical half, Z1-Z2 areas with measurements. (**e**) EDX analysis of the implant surface. (**f**) Percentage elemental analysis from the implant surface.

**Figure 2:**
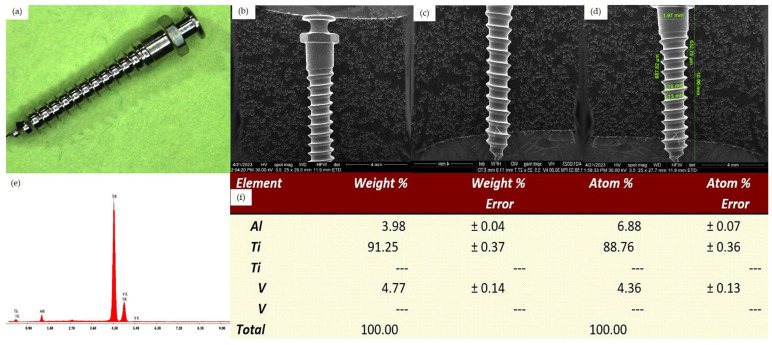
Surface analysis of an unused OrthAnchor System implant. (**a**) Overview aspect seen by optical microscopy, 10×. (**b**) SEM aspect of the cervical half, Z1-Z3 areas. (**c**) SEM aspect of the apical half, Z2-Z4 areas. (**d**) SEM aspect of the implant, Z2-Z4 areas with measurements. (**e**) EDX analysis of the implant surface. (**f**) Percentage elemental analysis from the implant surface.

For the two control groups samples, we could observe the surface morphology of new unused mini-implants, analyzed by optical microscopy, scanning electron microscopy and also the results of the EDX analysis. The 2 control groups correspond to the two mini-implants types used: control group 1 - Dual Top Anchor System implant (Figure 1) and control group 2 - OrthAnchor System implant (Figure 2).

*Study groups*

Group 1—infrazygomatic TAD

**Figure 3:**
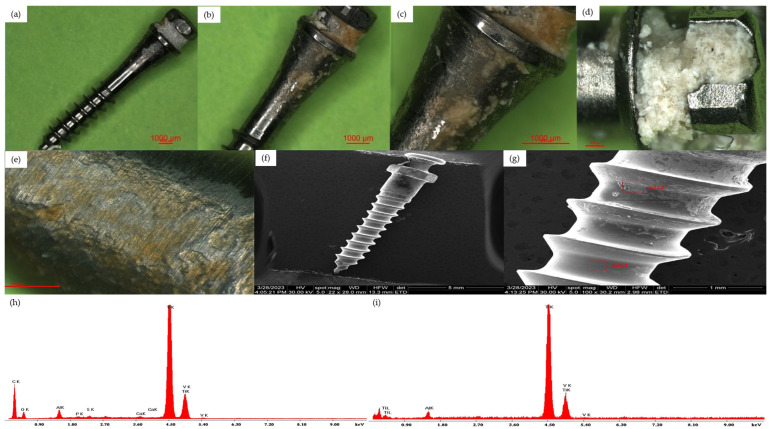
(**a**) Optical microscopy overview aspect of a Group 1 TAD, 10×. (**b**) Optical microscopy aspect of a Group 1 TAD, 15×; Z2—cervical surface. (**c**) Optical microscopy aspect of a Group 1 TAD, 30×; Z2—cervical surface. (**d**) Optical microscopy aspect of a Group 1 TAD, 45×; Z1—surface of the mini-implant head. (**e**) Optical microscopy aspect of a Group 1 TAD, 45×; Z2—cervical surface. (**f**) SEM overview aspect of a Group 1 TAD. (**g**) SEM aspect of the middle part Z3 area of a Group 1 TAD. (**h**) EDX analysis from the Z3 area of a Group 1 TAD surface. (**i**) EDX analysis from the Z4 area of a Group 1 TAD surface.

In the samples analyzed from the group of infrazygomatic TADs, we found massive deposits of bacterial plaque in most of the mini-implants on the exposed intra-oral surface all around the implant head, causing the retention of some foreign bodies, probably food, on the groove of the implant head, but also on the submucosal portion. In almost all samples, we encountered bacterial plaque deposits in the middle and apical thirds located inside the bone tissue, but in minimal quantities. In all samples, significant surface morphology changes of the mini-implant with the appearance of corrosion phenomena were observed. The EDX analysis revealed the presence of carbon in the areas covered with bacterial plaque deposits, proving their organic nature, whereas the clean surfaces had a similar composition to the control group TADs (Figure 3).

Group 2—interdental TAD

**Figure 4:**
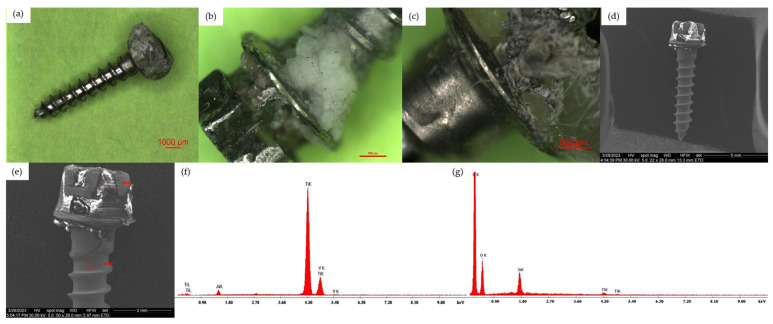
(**a**) Optical microscopy overview aspect of a Group 2 TAD, 10×. (**b**) Optical microscopy aspect of Group 2 TAD, 30×; Z1-Z2—cervical surface. (**c**) Optical microscopy aspect of Group 2 TAD, 45×; Z2—cervical surface. (**d**) SEM overview aspect of a Group 2 TAD. (**e**) SEM aspect of the head half, Z1-Z2 areas of a Group 2 TAD. (**f**) EDX analysis from the Z1 area of a Group 2 TAD surface. (**g**) EDX analysis from the Z2 area of a Group 2 TAD surface.

In order to increase the patient’s comfort as much as possible, for all interdental TADs in the maxilla or mandible, the head of the mini-implants were covered with acrylate (Triad Gel), and this further favored the deposition of bacterial plaque. The changes observed on the head of the mini-implant are due to the need to remove with a bur the acrylate deposited at this level at the time of mini-implant removal. The presence of acrylate and bacterial deposits on the TAD head was revealed by EDX analysis. Unlike infrazygomatic TADs, in the samples of interdental maxillary TADs, better represented bacterial plaque deposits were highlighted, as well as on all intraosseous threads in variable amounts right up to near the tip of the mini-implant. On the other hand, in the mandibular interdental TAD samples, the bacterial plaque was almost completely absent from the surface of the intraosseous coils in all cases analyzed (Figure 4). 

Group 3—palatal vault TAD

**Figure 5:**
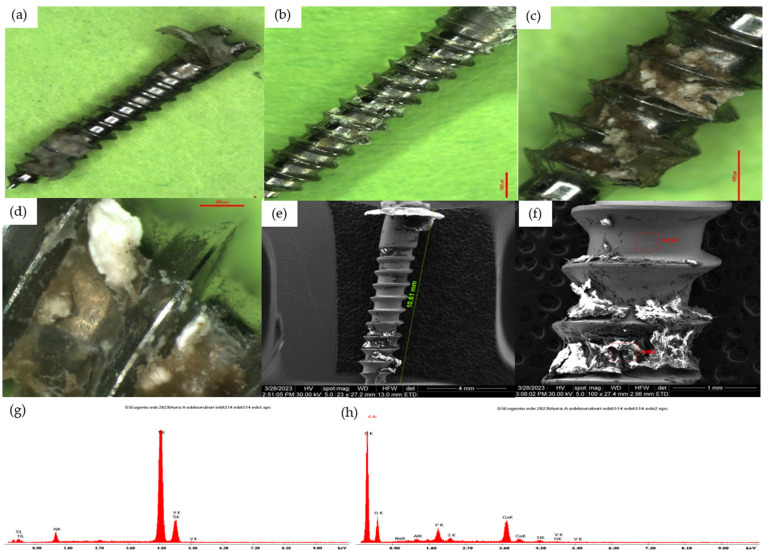
(**a**) Optical microscopy overview aspect of a Group 3 TAD, 10×. (**b**) Optical microscopy aspect of Group 3 TAD, 15×; Z2-Z4 areas of a Group 3 TAD. (**c**) Optical microscopy aspect of group 3 TAD, 30×; Z3-Z4 areas of a Group 3 TAD. (**d**) Optical microscopy aspect of Group 3 TAD, 75×; Z4 areas of a Group 3 TAD. (**e**) SEM overview aspect of a Group 3 TAD. (**f**) SEM analysis from the Z4 area of a Group 3 TAD surface. (**g**) EDX analysis of a clean zone from the Z4 area of a Group 3 TAD surface. (**h**) EDX analysis of a covered zone from the Z4 area of a Group 3 TAD surface.

The heads of the mini-implants in this group were completely covered with acrylate to avoid damaging the soft parts. Corrosion phenomena were observed in the mucosal surface of the mini-implants. The samples from this batch differ from the other samples in their depositions, especially in the middle third and at the junction of the middle third with the apical third of organic tissue and bacterial plaque remnants that modified the surface of the mini-implants, producing important corrosion phenomena. The organic nature of the observed deposits was validated by EDX analysis (Figure 5).

## 4. Discussion

We started our study with an analysis of the morphology of the studied implants, carried out before their introduction into the biological environment immediately after taking them out of the box. The morphology of the external surface of a mini-implant is important because the primary stability may be affected by parameters like length, diameter, and thread profiles [13,14], which justifies the permanent concern of the manufacturers for the identification of an optimal configuration [15].

In our analysis, we made observations of all TAD areas, starting with the TAD head. Its design has a special importance; its diameter is larger than the diameter of the transmucosal profile in order not to allow the soft tissues to cover it [16].

The increase in the number of threads and their shape determines the increase in the contact surface between the implant and the bone tissue, which will also determine the increase of the frictional force, of the primary stability and the resistance to movement [17].

In our study, measurements of the component elements of the TADs were made in order to be compared with the literature data. There is an increasing variety of orthodontic mini-implants available in different shapes, diameters, lengths, and titanium alloy compositions. Higher wire count and finer pitch in the mini-implant are associated with greater mechanical locking capacity, increased resistance during mini-implant placement, improved displacement resistance, and increased primary stability [18].

An important factor in the design of the mini-implant is its head, which must be wider than the transmucosal neck to prevent excessive soft tissue growth. Casaglia et al. showed that the small transmucosal diameter of the neck is a place of increased fragility [19].

Although titanium alloy orthodontic mini-implants present numerous advantages, practitioners must be aware of the topographical and microstructural characteristics of mini-implants, as they influence the effectiveness of orthodontic anchorage. A reliable and effective anchorage influences the orthodontic treatment. The primary stability, mechanical strength, and clinical performance of mini-implants in turn depend on their topographical and microstructural characteristics. The orthodontic mini-implants evaluated showed significant differences in the design of the screw head, transmucosal neck, threaded body, and active tip [20,21].

The results obtained in our study for the control group of new, unused TADs were similar to those identified in the specialized literature [22,23].

In our study, in all study groups, we found changes in the morphology of the implant surface, and especially deposits of biological material.

The biological oral environment is an aggressive habitat that affects the quality and surface of the implanted structures [24].

Surface modification is a constant challenge for dental implant manufacturers in the aim of a better acceptance and osseointegration [25,26,27]. However, in the case of TADs, one does not want osseointegration but rather their biological acceptance.

There are many factors that contribute to the degradation of the orthodontic mini-implant: chemical composition, surface morphology, the chemical composition of saliva, biofilm, the pH of the oral environment, protein adsorption, the physical and chemical properties of food, medications taken by the patient, and oral hygiene [28].

In our study, bacterial plaque deposits were identified in different areas in the study groups. The degradation of orthodontic mini-implants depends on the action of the oral environment while they are used for orthodontic treatment [29]. Biofilm is a surface film composed of different saliva components that are colonized with microorganisms in extracellular polymeric substances deposited on all surfaces in the oral cavity, including orthodontic appliances [30].

EDX analysis of the TADs’ surfaces verified that titanium is the main element found in their composition. Thanks to its attractive characteristics, for example, excellent biocompatibility and high corrosion resistance, titanium is also widely used in orthopedic components. It also presents excellent mechanical properties and offers resistance to stress and deformation; due to its excellent characteristics it is considered an ideal material [20,31,32].

Due to the high forces required to move the teeth, orthodontic mini-implants should withstand these loads. However, pure titanium has a lower fatigue strength than titanium alloys. During the placement and removal of orthodontic mini-implants, to overcome the potential fractures of pure commercial titanium, aluminum and vanadium have been added to the alloy to increase its strength and fatigue resistance [33,34,35], which makes this alloy the most used today in dentistry for this purpose [36].

A series of studies determined the elemental composition of the surface of mini-implants after their use in the biological environment and identified, in addition to the main elements, traces of Fe, S, and Ca, which probably came from bone tissue but also from blood [37,38]. In our study, EDX analysis showed the presence of carbon and oxygen in areas covered by bacterial plaque or areas covered by acrylate on the head of a used mini-implant. In other more recent studies [39], mini-implants extracted from patients were used, similar to those in our study, for a period of at least 8 months, and only elements similar to those in previous studies were identified. However, there were no concrete data related to a possible correlation between the amounts of additional elements deposited on the surface of the mini-implants and the time they were used in the biological environment. There is still a consensus that these observations do not recommend the reuse of micro-implants in other patients, and in general the possibilities of reuse must be better investigated [40,41,42,43,44].

Titanium is frequently used in the medicine of bone tissues due to its special properties, especially its excellent biocompatibility, but also its high resistance to corrosion [36].

In this study, EDX analysis showed that aluminum and vanadium were the main elements used in titanium alloying for the observed orthodontic mini-implants. In orthodontics, to improve the mechanical properties, pure titanium is alloyed with aluminum and vanadium to improve the resistance of the mini-implant, especially during placement and removal [45,46]. 

Although titanium alloys are considered very resistant to corrosion due to the stable passive layer of titanium oxide on their surface, they can no longer be considered inert [38,47]. In the oral cavity, aluminum and vanadium ions may be released in local tissues and can cause side effects in the human body [48]. The corrosion resistance of orthodontic alloys depends on the oral environment, which is influenced by several variables, such as the amount and quality of saliva and pH of food and beverages, among others [49].

Moreover, in our study we observed corrosion surfaces on the TADs from all three study groups. A factor that contributes to the inflammation of biological tissues is the corrosion of orthodontic mini-implants or temporary anchorage devices (TADs), which, in turn, is also a factor in the clinical success of mini-implants, since we do not want total, but partial, osseointegration. The reasons why a mini-implant can induce inflammation of the surrounding biological tissues are multifactorial, including the level of hygiene of the patient, the type of surrounding tissue [50], and the design of the mini-implant head, but it should be noted that in some studies, macrophages loaded with titanium resulting from the corrosion process have been observed around failed orthodontic mini-implants [51,52].

The corrosion properties of orthodontic mini-implants have only been examined in two ion release studies [53]. Morais et al. [35] used a rabbit model and detected traces of titanium, aluminum, and vanadium from orthodontic mini-implants in distant organs.

An interesting future study could include corrosion analysis. Oxygen reacts with the surface titanium to form a clear oxide, TiO_2_. Depending on the thickness of the oxide, the color of the alloy is modified. If the thickness of the oxide is increased, the interaction with light is modified, and the titanium material (or alloy) appears colored red. If the oxide layer is thinner, then the color of the alloy changes to silver/grey. Thermal and electrochemical treatments are common methods to modify the thickness of the oxide layer [54].

As we mentioned before, an increase in the corrosion extent can affect the properties of titanium-based mini-implants [55], and the release of elements from the mini-implants has the potential to alter the inflammatory process, increasing the chances of complication or failure [56].

Metal surfaces without biofilm have access to oxygen from the outside while metal surfaces covered by biofilm are protected from contact with oxygen from the outside. The difference in oxygenation creates a corrosive cell, and the metal under the biofilm becomes the anodic site, allowing electrons and thus corroding. From the anodic site, the electrons reach the metal surface freely accessible to oxygen, where they form hydroxyl ions. This is the mechanism by which microorganisms generally contribute to corrosion through the formation of biofilms and oxygen concentration corrosive cells [57].

## 5. Conclusions

The biological environment is aggressive for mini-implant structures, always producing changes to their surface during their clinical use. On all samples examined after clinical use, regardless of the period of use, corrosion surfaces in different locations were observed. The areas of the mini-implants exposed in the oral cavity were more affected both due to the direct impact and the need to apply acrylic materials to protect the soft parts, which favors bacteria deposition. The areas of corrosion identified at the level of the intraosseous portions of the mini-implants vary depending on the area of their insertion, being more frequent in the cervical third, especially for mini-implants inserted in the infrazygomatic area, disposed on a wider surface towards the apical third for the interdental mini-implants and more concentrated in the junction of the medial third with the apical third in the case of the mini-implants inserted in the palatal vault.

## Data Availability

The authors declare that the data of this research is available from the corresponding authors upon reasonable request.
